# New and old criteria for diagnosing celiac disease: do they really differ? A retrospective observational study

**DOI:** 10.1186/s13052-024-01625-w

**Published:** 2024-04-01

**Authors:** Salvatore Accomando, Ilenia Rita Piazza, Francesca Cacciatore, Veronica Notarbartolo, Giovanni Corsello, Mario Giuffrè

**Affiliations:** 1 Paediatrics Operative Unit, Department of Health Promotion, Mother and Child Care, Internal Medicine and Medical Specialties (PROMISE), School of Medicine, University of Palermo, Palermo, Italy; 2 Department of Health Promotion, Mother and Child Care, Internal Medicine and Medical Specialties (PROMISE), School of Medicine, University of Palermo, Palermo, Italy

**Keywords:** Celiac Disease, ESPGHAN Criteria, Autoimmune Associated disorders

## Abstract

**Background:**

The aim of this study is to compare two groups of celiac patients: the first one, in which diagnosis was based on a “biopsy sparing” approach according to the 2012 ESPGHAN criteria, and the second one, based on the biopsy approach like the one of the 1991 Revised Criteria, in order to find relevant difference for sex, M/F *ratio*, age at diagnosis, clinical features at the onset, presence and prevalence of concomitant autoimmune disorders.

**Methods:**

Our study involves 61 patients having the Celiac Disease (CD) onset from February 2013 to February 2020. The 32 patients who received diagnosis according “biopsy sparing” criteria were enrolled in group (1) The 29 patients who received diagnosis by duodenal biopsy were enrolled in group (2) Prevalence of comorbidities was analysed through chi-square test.

**Results:**

In group 1 the prevalence of comorbidities such as Insulin-Dependent Diabetes Mellitus (IDDM) and thyroiditis was of 53%, while in group 2 it was only of 24%. Analysing the IDDM prevalence between the two groups we found a relevant difference. At the same time, the prevalence of thyroiditis was also significantly different. In group 1, male patients, in particular, would seem to have a higher incidence of CD related autoimmune disorders.

**Conclusions:**

An increased prevalence of IDDM, thyroiditis and juvenile idiopathic arthritis (JIA) in the first group would show that the “biopsy sparing” approach could expose patients to a greater length of disease activity that might be responsible for the onset of such comorbidities. Further studies should be carried out on more numerous samples of patients in order to confirm or not these data.

**Supplementary Information:**

The online version contains supplementary material available at 10.1186/s13052-024-01625-w.

## Background

Celiac Disease (CD) is an immune-mediated systemic disorder elicited by gluten and related prolamins in genetically susceptible individuals and characterised by the presence of a variable combination of gluten-dependent clinical manifestations, CD-specific antibodies, HLA-DQ2 or HLA-DQ8 haplotypes, and enteropathy. CD-specific antibodies comprise autoantibodies against transglutaminase (TTG), including anti-endomysium antibodies (AEA), and antibodies against deamidated gliadin peptides (DGP). CD may present with a large variety of nonspecific signs and symptoms. It is important to diagnose CD not only in children with obvious gastrointestinal symptoms but also in children with a less clear clinical picture because the disease may have negative health consequences, involving several extra-intestinal organs (i.e. development of other autoimmune disorders, such as type 1 diabetes and autoimmune thyroid disease; multiple sclerosis; anaemia, osteopenia, neurological conditions like epilepsy and migraine). The availability of serological tests with high accuracy and other, more invasive, diagnostic test like duodenal biopsy, allows a firm diagnosis to be made. Testing for CD should be offered to children and adolescents with the otherwise unexplained symptoms and signs of chronic or intermittent diarrhoea, failure to thrive, weight loss, stunted growth, delayed puberty, amenorrhoea, iron-deficiency anaemia, nausea or vomiting, chronic abdominal pain, cramping or distension, chronic constipation, chronic fatigue, recurrent aphthous stomatitis, dermatitis herpetiformis or fracture with inadequate traumas/osteopenia/osteoporosis, and abnormal liver biochemistry. In addition, children with type 1 diabetes mellitus (T1DM), Down syndrome, autoimmune thyroid disease, Turner syndrome, Williams syndrome, selective immunoglobulin A (IgA) deficiency, autoimmune liver disease, juvenile idiopathic arthritis (JIA) or *vitiligo* are at increased risk for CD. CD is often associated with one or more of the abovementioned conditions, therefore these children should be periodically tested for CD [1]. In particular, recent data [2] show that the estimated prevalence of CD in IDDM affected patients is about 6%, and about 1% in the general population. At the same time, an association between CD and AITD (Auto-Immune Thyroid Diseases) has been demonstrated in variable percentages between 2% and 7.8%, 3 times higher than in general population [3].

In 2012 with the availability of a CD specific serology consisting of anti DGP, AEA and anti TTG antibodies in association with the fact that almost 100% of CD subject have DQ2 or DQ8 HLA antigens, times were mature for a “biopsy sparing” approach. So, the ESPGHAN CD task force approved the diagnosis of the condition without biopsy, proved that certain conditions should be satisfied. In 2019 the same task force accepted the matter of fact that DQ2 or DQ8 HLA are useful for extremely high negative predictive value and then they are not necessary to confirm diagnosis [4].

Patients with positive anti-TTG antibody levels lower than 10 times Upper Normal Level (UNL) given by the manufacturer of this particular test should undergo upper endoscopy with multiple biopsies. On the other hand, the paediatric gastroenterologist should discuss with the parents and the patient who is positive for anti-TTG antibody levels > 10 times UNL (as appropriate for age) the option of omitting the duodenal biopsies and the implications of doing so. If the parents or the patient accept this option, then blood should be drawn for HLA and AEA testing. It is important that AEA testing be performed from a different blood sample than anti-TTG testing to exclude false-positive results because of mislabelling of the previous sample or other mistakes in processing and reporting. If the patient tests positive both for AEA antibodies and for HLA-DQ2 or HLA-DQ8, the diagnosis of CD is confirmed. A Gluten Free Diet (GFD) is started and the patient is studied for improvement of symptoms and decline of antibodies. A later gluten challenge in these children is not required or recommended [5].

### Aims

The aim of our study is to compare two groups of patients: the first one in which diagnosis of CD was based on 2012, “biopsy sparing” ESPGHAN Criteria, and the second one based on 1991 Revised ESPGHAN Criteria. The objective of the study is to find relevant differences between the two groups according to the examined variables: sex, M/F *ratio*, age at diagnosis, clinical features at the onset, presence and prevalence of concomitant autoimmune disorders.

## Patients and methods

In the seven-year period going from February 2013 to February 2020, 61 patients diagnosed with CD were recruited. Patients whose diagnosis was performed according to the “biopsy sparing” 2012 Criteria were enrolled in the group (1) The patients who received diagnosis according to the Revised 1991 ESPGHAN Criteria and who underwent duodenal biopsy were enrolled in the group (2) All patients were tested for Anti TTG IgA antibodies and deamidated AGA serology performed with the enzyme-linked immunosorbent assay (ELISA) method. AEA were tested in all subjects with an indirect immunofluorescence method. The total number of IgA immunoglobulins and HLA were also tested. DQ2 and/or DQ8 were performed in all patients. Patient recruitment was performed using data collected in our Paediatric Gastroenterology Unit. The comorbidities were identified through individual follow-up plans. The first group included patients who had Anti TTG IgA Antibodies > 10 Times UNL, who avoided duodenal biopsy and underwent a new blood AEA sampling. The second group included patients with Anti TTG IgA Antibodies < 10 Times ULN. The patients in the second group underwent duodenal biopsy with multiple sampling: five biopsies including four samples of second duodenal portion and one sample of duodenal bulb.

The diagnosis of autoimmune thyroiditis was made through the detection of antibodies to thyroid peroxidase (Ab anti-TPO) and anti-thyroglobulin (Ab anti-Tg) in the blood, and with the ultrasound evidence of uneven thyroid and/or hyper echoic pattern [6].

IDDM identification was performed according to the ISPAD criteria [7–8].

All patients with IDDM were screened for CD with deamidated gliadin and tissue transglutaminase antibody testing, following the autoimmune disease screening policy.

A definitive epidemiological association between CD and JIA has been established. According to the recent literature, > 2.5% patients with JIA were diagnosed with CD and, at the same time, the CD prevalence in JIA patients may be even higher (> 3–3.5%) [9]. The most common symptoms of JIA are joint inflammation, swelling and tenderness of the joints, fatigue, joint stiffness, muscular weakness and, in the systemic form (sJIA), also known as Stills’ disease, fever, skin rash and enlargement of the lymph nodes [10]. The diagnosis of JIA is based on the International League of Associations for Rheumatology (ILAR) criteria [11, 12].

Informed consent for blood sampling or undergoing duodenal biopsy, at time of diagnosis, was obtained from each patient, or his/her parent, involved in this study.

The study protocol conforms to the ethical guidelines of 1975 Declaration of Helsinki (6th revision, 2008).

### Statistical analysis

Prevalence of comorbidities was analysed through chi-square test. All significance tests were two-tailed, and *p* < 0.05 was considered significant. Statistical analysis was carried out through Microsoft Excel 2010.

## Results

**Fig. 1:**
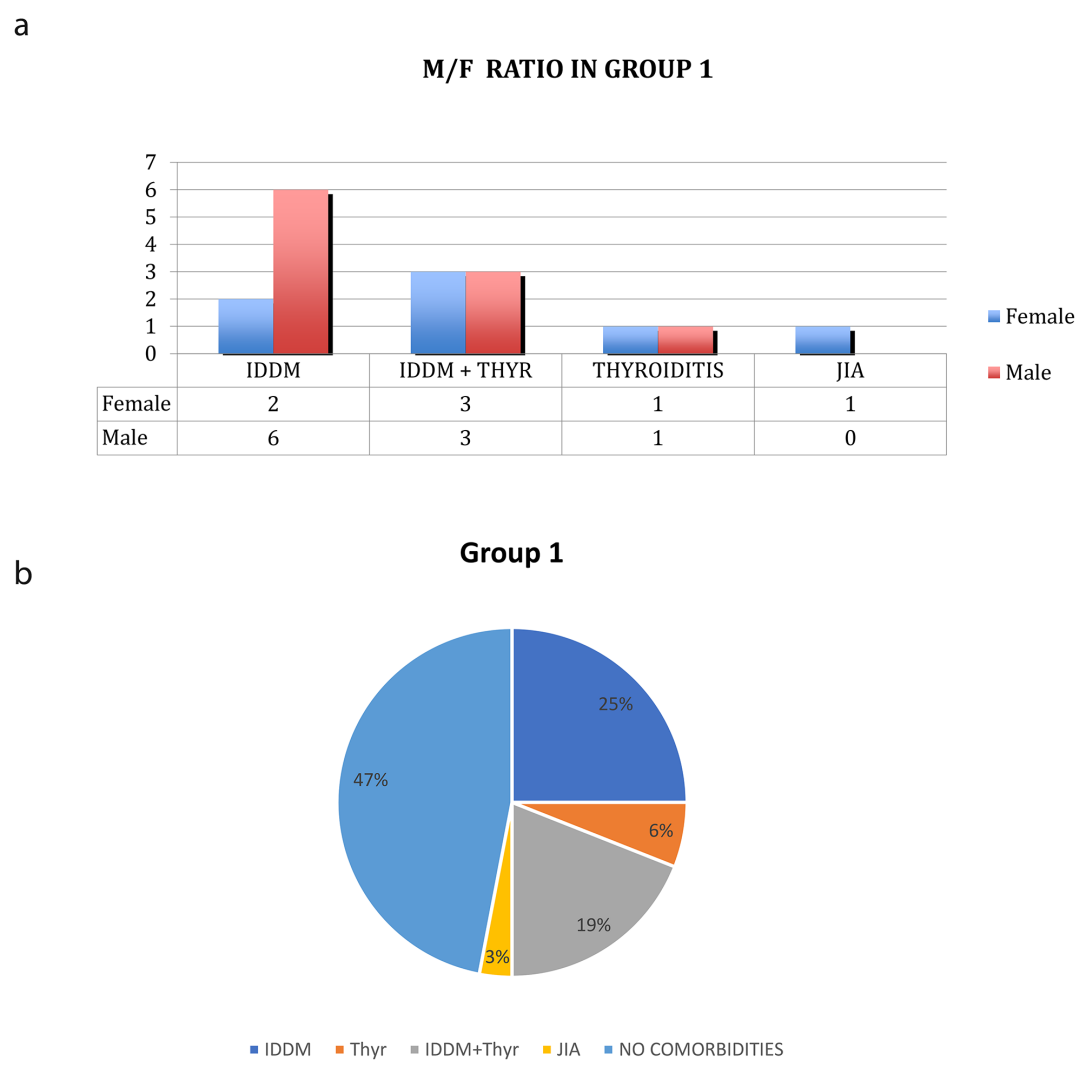
**a-b.** Sex distribution (M/F *ratio*) related to prevalence of autoimmune disorders in group 1. In this group, there was a statistically significant difference in prevalence for IDDM (P of χ ^2^ 0.05) and for autoimmune thyroiditis (P of χ ^2^ 0.01)

**In group 1**, the diagnosis was performed according to the 2012 ESPGHAN Criteria. This group included **32** patients: 14 males (M) and 18 females (F). 17 of them had associated comorbidities: 10 M and 7 F. (see Fig. 1a). In this group there was a prevalence of comorbidities equal to 53% (see Fig. 1b): 8 patients (6 M/2F) had IDDM, 6 patients (3 M/3F) had both IDDM and thyroiditis, 2 patients (1 M/1F) had thyroiditis, and 1 patient (F) had oligo articular JIA. One male patient was affected by Down Syndrome. The average age of the group at the time of diagnosis was 7,25 years ± 3,91.

**Fig. 2:**
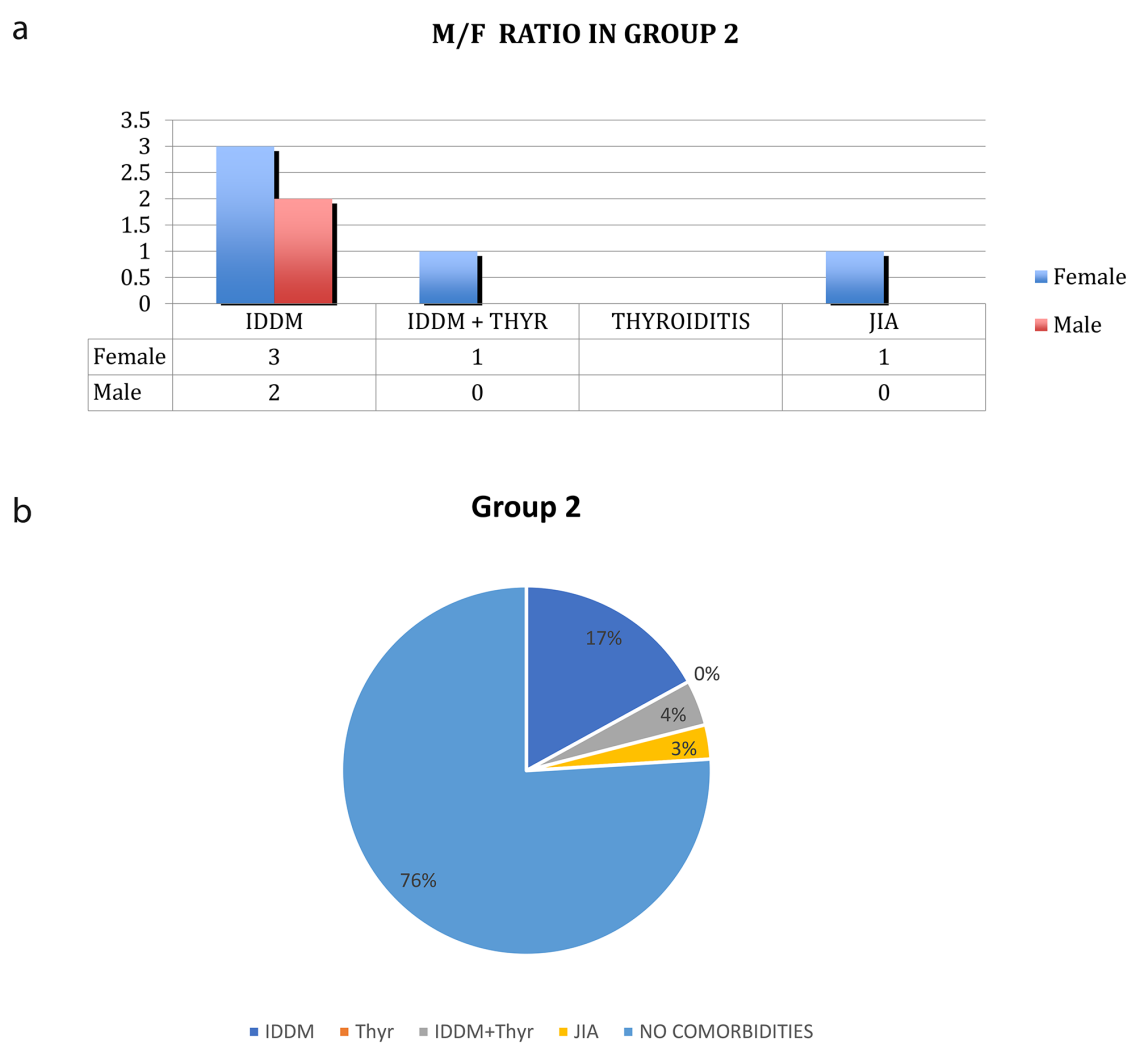
**a-b.** Sex distribution (M/F *ratio*) related to prevalence of autoimmune disorders in group 2. Statistical analysis documented increased prevalence of autoimmune disorders in the first group of CD patients

**Fig. 3:**
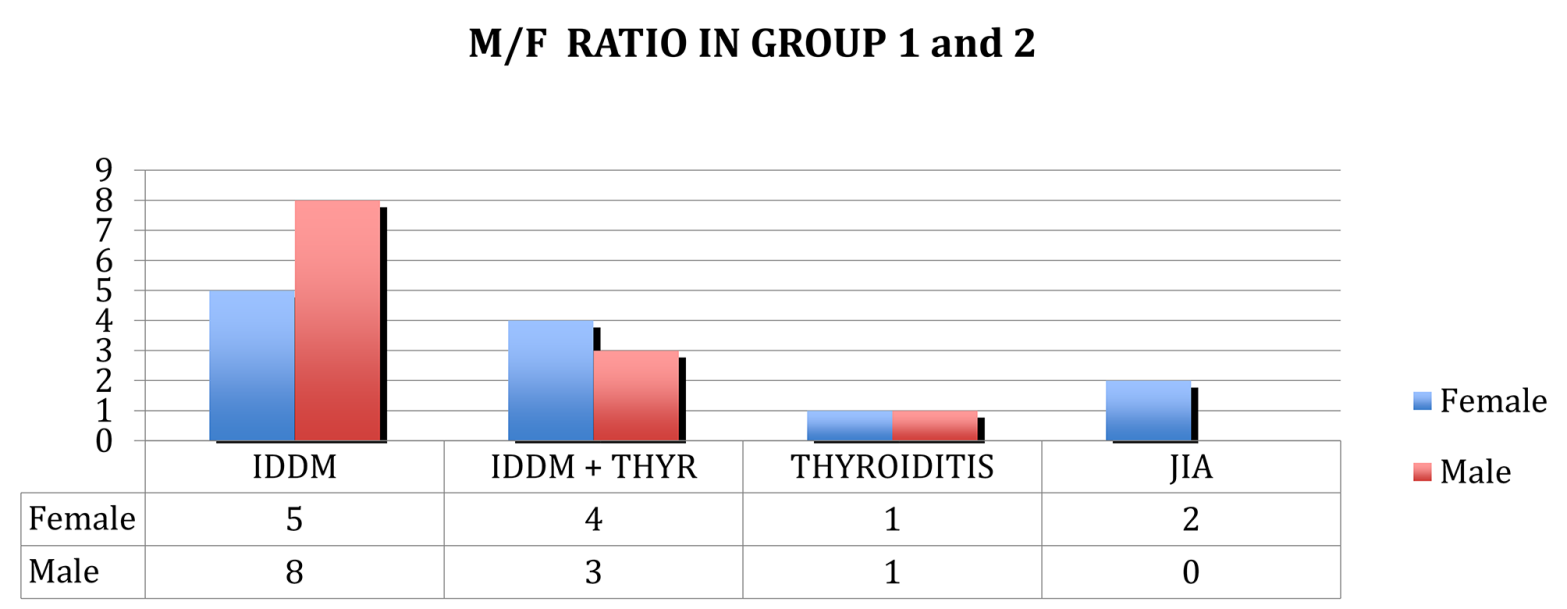
Sex distribution (M/F *ratio*) related to prevalence of autoimmune disorders in group 1 and 2

**In group 2**, the diagnosis was performed according to the Revised 1991 ESPGHAN Criteria. This group included **29** patients: 10 M and 19 F. 7 of them had associated autoimmune diseases: 3 M and 4 F. (see Fig. 2a). In this group there was a prevalence of comorbidities equal to 24% (see Fig. 2b): 5 patients (3 M/2F) had IDDM, 1 patient (F) IDDM and thyroiditis, 1 patient (F) had oligo articular JIA. One patient was affected by Turner Syndrome. The average age of the group at the time of diagnosis was 7.12 years ± 4,59. In Fig. 3, a sex distribution related to prevalence of autoimmune disorders in the two groups.

Statistical analysis documented increased prevalence of autoimmune disorders in the first group of CD patients. The *P* value of χ ^2^ test was indeed: 0.02. Analysing the difference of the IDDM prevalence between the two groups of patients, we found a *P* value of χ ^2^ test of 0.05. Likewise, also the difference in prevalence for autoimmune thyroiditis was significant with the *P* value of χ ^2^ test of 0.01.

Although in group 1 the prevalence of male patients who had concomitant disorders was greater than group 2, there was not a statistically significant difference between the two groups in M/F *ratio*.

**Fig. 4:**
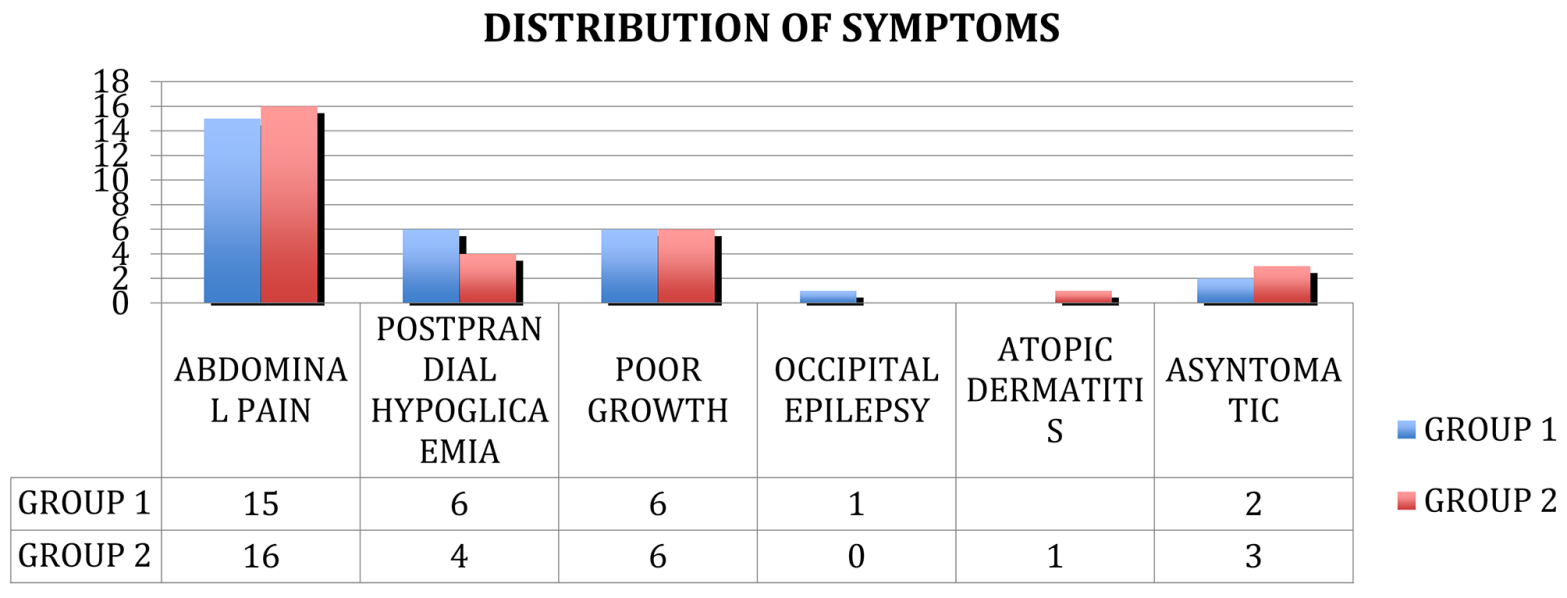
Symptoms distribution at celiac disease onset in the two groups

The main onset symptom of CD in the first group was recurrent abdominal pain present in 15 (7 M/8F) of them, representing 48% of all group patients. The other way of onset of CD was poor growth associated with stools’ alteration in 19% of cases (6pts: 2 M/4F), 2 female patients had anaemia, 2 patients were asymptomatic, 1 female patient reported occipital epilepsy (see Fig. 4).

The main onset symptom of CD in the second group was recurrent abdominal pain in 55% of patients, 16 (8 M/8F) of them. Poor growth was observed in 20% of cases, 6 (3 M/3F) associated with hypoglycaemia in 4 patients (3 M/1F) with IDDM, 3 patients (2 M/1F) had microcytic anaemia and 2 (1 M/1F) of them had stool frequency disorders (mainly constipation), 3 male patients were asymptomatic and 1 patient (F) with poor growth had also atopic dermatitis associated with a high IgE title ( see Fig. 4).

## Discussion

Analysis of our study data showed that the female sex is prevalent among the 2 groups. In fact, CD was more prevalent in female sex, as also reported in literature.

However, there was a significant difference in the distribution between the two sexes about the prevalence of autoimmune comorbidities (type I diabetes mellitus, thyroiditis and JIA). In fact, in the first group the male sex was the one who was more affected by autoimmune comorbidities, while the female sex was more affected in the second group. Since the prevalence of female sex in autoimmune diseases was well known in literature, in particular in thyroiditis [13], it was probably a longer time from disease onset that determined a higher prevalence of autoimmune comorbidities in our group 1, which in our study mainly involved males. The prevalence percentages of autoimmune diseases found in this group are comparable to those present in the literature.

In particular, the development of IDDM in patients with celiac disease was more frequent in males than in females in both our groups. This finding seems to agree with the higher prevalence of male sex in IDDM population.

The single analysis of comorbidity “thyroiditis” showed a significant difference with a *P* value of 0.02 between the two groups confirming that a longer time since disease onset increased the risk of comorbidity and this affected male and female sex equally (4 M and 4 F) in the first group. In the second group, only one female child had thyroiditis and celiac disease as more frequently reported in literature.

Also, patients affected by oligo articular JIA were females in both groups according to literature data [14].

About the average age at diagnosis there is no significant difference between the two groups, however children belonging to the “biopsy sparing” group may have longest time of disease onset needed to rise the anti-TTG IgA levels to ten times UNL.

Although there was no difference in the age of onset between the two groups, it was more probably that the disease in the subjects belonging to the first group has arisen before the clinical onset.

The analysis of symptoms at the onset of celiac disease in the two groups showed no significant differences. In fact, recurrent abdominal pain was the most important symptom in both groups, followed by postprandial hypoglycaemia in patients with IDDM and then by delay of somatic growth. Recurrent hypoglycaemia, in particular postprandial hypoglycaemia, was also increased in IDDM and CD (see Fig. 4). Recurrent hypoglycaemic events can lower the hypoglycaemic threshold at which symptoms occur in an individual, due to attenuation of the sympathoadrenal response, and may influence brain development and function [15–16]. In the first group one male patient presented Down Syndrome. Feeding problems and gastrointestinal disorders are the most common anomalies in people with Down syndrome [17] and have a significant impact on their daily life: in particular for celiac serology there is no consensus on the starting age and the frequency of screening, but numerous studies propose every year from the age of 2 years.

The risk of autoimmune diseases was approximately twice as high in females who had Turner Syndrome [18] compared to the general female population. The spectrum included Hashimoto’s thyroiditis, CD, IDDM, alopecia areata, inflammatory bowel disease, juvenile rheumatoid arthritis and so on [19]. In this study only one patient was affected by Turner Syndrome and CD with no other comorbidities.

In literature, the rates of extraintestinal manifestations of CD are similar in adults and in children. In children, short stature, fatigue, and headache are more represented [20]. Amongst the neurological manifestations, cerebellar ataxia, peripheral neuropathy and epilepsy, have been recognized as possible complications and/or starting manifestation of CD [21]. In our female patient the neurological manifestation, started at age of nine, was represented by an occipital epilepsy form associated with abdominal pain and anaemia. Patients with CD frequently develop antibodies against antigens related to the endocrine organs and in the same way, some authors described autoantibodies acting as possible “triggers” of neurological manifestations. Also, the risk for developing neurological manifestations, as for other autoimmune disorders seems higher in case of long-standing gluten exposure. In our case the neurological manifestation disappeared after a gluten free diet, according to the hypothesis that an important role could be played by the duration of gluten exposure [22–24].

The data analysis showed that four patients, in group 1, have IgA anti TTG levels upper to 30 times the norm, but only one female patient of these presents IDDM. According to our findings, extremely elevated levels of IgA anti TTG do not appear to have increased associated comorbidities in the same patients. A higher title of antibodies does not seem to correlate directly with a higher risk of comorbidity, even if it is related with a high damage in enteropathy [25].

## Conclusions

An increased prevalence of overall comorbidities (IDDM and thyroiditis) in the first group shows that the new diagnostic criteria could expose patients to a long-lasting gluten intake that may be responsible for the onset of such comorbidities. Nevertheless, not all patients with the highest levels of anti TTG antibodies present associated autoimmune conditions. It would be interesting to evaluate if there is a specific genetic disease subset in this group (may male gender has a role?) that is more frequently associated with an increased risk of comorbidities. The limited number of the study population does not permit to evaluate if male gender may have a role in this differentiation. Since that the 2012 ESPGHAN criteria could expose male celiac patients to more relevant incidence of associated pathological conditions so, it is particularly important to reduce diagnostic delay and to pay attention to the onset of the autoimmune associated conditions in this sub group of male celiac patients. Further studies should be carried out on more numerous groups of patients to highlight possible statistically significant differences between the two different ways of diagnosis, also in order to find out other genetical factors that may play a role in the different disease behaviour, natural history and clinical association with other autoimmune conditions of celiac disease.

### Electronic supplementary material

Below is the link to the electronic supplementary material.

Supplementary Material 1

Supplementary Material 2

## Data Availability

Data and materials were collected from medical records and are available from the corresponding author on reasonable request.

## Abbreviations

CD: Celiac Disease
TTG: Trans glutaminase
AEA: Anti Endomysium Antibodies
DGP: Deamidated Gliadin Peptides
IDDM: Insulin Dependent Diabetes Mellitus
JIA: Juvenile Idiopathic Arthritis
GFD: Gluten free diet Upper Normal Levels
