# Supplementary material for: New and old criteria for diagnosing celiac disease: do they really differ? A retrospective observational study
Source: Ital J Pediatr. 2024 Apr 1;50:59. doi: 10.1186/s13052-024-01625-w (PMC10986084; doi:10.1186/s13052-024-01625-w)
Supplement: Supplementary file 1 — Supplementary Material 1 [file 13052_2024_1625_MOESM1_ESM.pdf]

SPRINGER NATURE

Change of authorship request form - Journals (pre-acceptance)

Section 5: Author contribution, Acknowledgement and Disclosures. Please use this section to provide a new disclosure statement and, if appropriate, acknowledge any contributors who have been removed as authors and ensure you state what contribution any new authors made (if applicable per the journal or book (series) policy). Please ensure these are updated in your manuscript - after approval of the change(s) - as our production department will not transfer the information in this form to your manuscript.

New acknowledgements:

New Disclosures (financial and non-financial interests, funding):

New Author Contributions statement (if applicable per the journal policy):

A NEW AUTHOR WAS BROUGHT IN AND HE TOOK CARE OF THE REVISION OF THE TEXT.

State 'Not applicable' if there are no new authors.

SPRINGER NATURE Springer Nature is one of the world's leading global research, educational and professional publishers, created in May 2015 through the combination of Nature Publishing Group, Palgrave Macmillan, Macmillan Education and Springer Science+Business Media.

SPRINGER NATURE   Change of authorship request form - Journals [illegible] - Journ

Section 6: Declaration of agreement. *All* authors, unchanged, new and removed *must* sign this declaration.
(NB: Please print the form, (docu)-sign and return/upload a scanned copy. Please note that signatures that have been inserted as an image file are acceptable as long as it is handwritten. Typed names in the signature box are unacceptable.) *** Please delete as appropriate. Delete all of the bold if you were on the original authorship list and are remaining as an author.**

	First name	Family name		Signature	Date
1st author	SALVATORE	ACCOMANDO (CA)	I agree to the proposed new authorship shown in section 4 **/and the addition/removal*of my name to the authorship list** /and the proposed change in corresponding author	Salvatore Accomando	28/12/2023
2nd author	MARIA RITA	PIAZZA	I agree to the proposed new authorship shown in section 4 **/and the addition/removal*of my name to the authorship list** /and the proposed change in corresponding author	Maria Rita Piazza	28/12/2023
3rd author	FRANCESCA	CACCIATORE	I agree to the proposed new authorship shown in section 4 **/and the addition/removal*of my name to the authorship list** /and the proposed change in corresponding author	Francesca Cacciatore	28/12/2023
4th authors	VERONICA	NOTARBARTOLO	I agree to the proposed new authorship shown in section 4 **/and the addition/removal*of my name to the authorship list** /and the proposed change in corresponding author	Veronica Notarbartolo	28/12/2023
5th author	GIOVANNI	CORSELLO	I agree to the proposed new authorship shown in section 4 **/and the addition/removal*of my name to the authorship list** /and the proposed change in corresponding author	[illegible]	28/12/2023
6th author	MARIO	GIUFFRE'	I agree to the proposed new authorship shown in section 4 **/and the addition/removal*of my name to the authorship list** /and the proposed change in corresponding author	[illegible]	28/12/2023
7th author			I agree to the proposed new authorship shown in section 4 **/and the addition/removal*of my name to the authorship list** /and the proposed change in corresponding author		

SPRINGER NATURE

Change of authorship request form - Journals (pre-acceptance)

Section 1: Please provide the current title of manuscript

Manuscript ID no.: ITSP-D-22-00576

Title: NEW AND OLD CRITERIA FOR DIAGNOSING CELIAC DISEASE: DO THEY REALLY DIFFER? A RETROSPECTIVE OBSERVATIONAL STUDY

Section 2: Please provide the previous authorship, in the order shown on the manuscript before the changes were introduced. Please indicate the corresponding author by adding (CA) behind the name.

	First name(s)	Family name	ORCID or SCOPUS id, if available
1st author	SALVATORE	ACCOMANDO (CA)	
2nd author	ILENIA RITA	PIAZZA	
3rd author	FRANCESCA	CACCIATORE	
4th author	GIOVANNI	CORSELLO	
5th author	MARIO	GIUFFRE'	
6th author			
7th author			
8th author			
9th author			
10th author			

Please use an additional sheet if there are more than 10 authors.

SPRINGER NATURE Springer Nature is one of the world's leading global research, educational and professional publishers, created in May 2015 through the combination of Nature Publishing Group, Palgrave Macmillan, Macmillan Education and Springer Science+Business Media.

SPRINGER NATURE

Change of authorship request form - Journals (pre-acceptance)

Section 3: Please provide a justification for change. Please use this section to explain your reasons for changing the authorship of your manuscript, e.g. what necessitated the change in authorship? Please refer to the (journal) policy pages for more information about authorship. Please explain why omitted authors were not originally included and/or why authors were removed on the submitted manuscript.

A NEW AUTHOR HAS BEEN BROUGHT IN TO REVIEW THE TEXT.

Section 4: Proposed new authorship. Please provide your new authorship list in the order you would like it to appear on the manuscript. Please indicate the corresponding author by adding (CA) behind the name. If the Corresponding Author has changed, please indicate the reason under section 3.

	First name(s)	Family name (this name will appear in full on the final publication and will be searchable in various abstract and indexing databases)	Affiliated institute	E-mail address
1st author	SALVATORE	ACCOMANDO (CA)	DEPARTMENT OF [illegible] AND CHILD CARE (PROMISE), SCHOOL OF MEDICINE, UNIVERSITY OF PALERMO.	salvatore.accomando@unipa.it
2nd author	ILENIA CLITA	PIAZZA	" "	[illegible]@gmail.com
3rd author	FRANCESCA	CACCIATORE	" "	francesca.cacciatore[illegible]@gmail.com
4th author	VERONICA	NOTARBARTOLO	" "	veronica.notarbartolo@gmail.com
5th author	GIOVANNI	CORSELLO	" "	giovanni.corsello@unipa.it
6th author	MARIO	GIUFFRE'	" "	mario.giuffre@unipa.it
7th author				
8th author				
9th author				
10th author				

Please use an additional sheet if there are more than 10 authors.

SPRINGER NATURE Springer Nature is one of the world's leading global research, educational and professional publishers, created in May 2015 through the combination of Nature Publishing Group, Palgrave Macmillan, Macmillan Education and Springer Science+Business Media.
