# Supplementary material for: New and old criteria for diagnosing celiac disease: do they really differ? A retrospective observational study
Source: Ital J Pediatr. 2024 Apr 1;50:59. doi: 10.1186/s13052-024-01625-w (PMC10986084; doi:10.1186/s13052-024-01625-w)
Supplement: Supplementary file 2 — Supplementary Material 2 [file 13052_2024_1625_MOESM2_ESM.docx]

**Point by point reply letter**

Dear Sirs,

In order to improve our manuscript number ITJP-D-22-00576, we made these modifications according to the changes suggested by the reviewers:

**R:** We believe that referring to the diagnostic guidelines as a strategy to cluster the patients into two groups is not correct. The diagnostic approach provided by those guidelines should rather be used, and the two groups better identified based on a "biopsy" vs "non-biopsy" approach for diagnosis of celiac disease. In fact, the latest (2020) diagnostic guidelines include each of the two approaches, and this is the case also for the 2012 guidelines that the authors refer to.

-The two groups were identified, according to the referee’s suggestion, in “Biopsy group” *vs* “Non-biopsy group”.

**R:** In order to truly compare the prevalence of autoimmune comorbidities between the two groups, the time of follow-up should be included in the analysis as a longer the follow-up may be associated with higher chances of an additional autoimmune disease.

-This is a retrospective, observational study, so we can only assess the prevalence of autoimmune comorbidities at CD onset, comparing the two groups at that moment. It would be interesting, as suggested by the referee, having additional data on a follow-up period, but we focused on and collected data only at celiac disease onset for both groups.

**ABSTRACT**

**R**: Aims should be better clarified. Both in the abstract ad in the introduction, it should be clearly stated whether the aim is to describe differences between the two cohorts in terms of clinical and epidemiological data or to assess the differences between the two ESPGHAN diagnostic guidelines. In addition, the term "new" to the 2012 diagnostic guidelines should probably be avoided.

-Aims were better clarified assessing that we compared two groups referring to them as biopsy group *vs* “biopsy sparing” group. The term “new” was deleted.

**R**: Methods: please indicated how many patients are in each group even in the abstract

-The number of patients for each group was added.

**R**: Results: should be better reported and the emphasis in the conclusions should be a little reorganised, lowering the tones of the findings.

-Results were modified and the conclusions reorganized lowering the tones of findings.

**R**: Title - it should probably incapsulate better the main results.

-It was modified pointing out a question if there is a real difference between the two ways of diagnosis.

**INTRODUCTION**
**R:** Line 7, please consider using "deamidated gliadin peptides" instead of " deamidated forms of gliadin peptides".

-“Deamidated Gliadin Peptides” was used instead of “Deamidated forms of gliadin peptides”.

**R:** Line 10, please clarify what type of clinical health consequences

-Some clinical health consequences were added.

**R:** Line 26, please avoid using "committee" referring to the ESPGHAN task-force engaged in the guidelines.

-“Task force” substituted the term “committee”.

**PATIENTS AND METHODS
R:** Line 48, please clarify the exact time frame of enrolment, considering also that the diagnostic criteria chosen were from 1991 and 2012.

-Time of enrolment was specified.

**R:** Line 65-66, this could be avoided, there is no need to clarify this point. Lines 67-76, this whole paragraph seem to me not relevant as the diagnosis is made according to the ISPAD criteria, a sentence including this and a reference should suffice.

-They were modified according to referee suggestions, referring to ISPAD criteria and erasing some not relevant sentences.

**R:** Lines 79-85, this paragraph needs also editing. Did the authors consider only the diagnosis of systemic JIA (they refer to sJIA). This is unlikely, as sJIA represents only a lower number of cases of JIA.

- sJIA was substituted by JIA that is more common also in our survey.

**R:** Line 86, a reference number for the Ethical Committee approval needs to be indicated, if the committee gave approval. However, in the declarations the authors say that retrospective studies do not require approval, please be consistent and provide formal evidence as of what is the rule for your Institution.

- Retrospective, observational studies, without neither pharmacological use, nor active investigator’s role (id est group randomization), are exempted from being approved by Palermo 1 ethics committee.

**R:** In the introduction the prevalence of T1D AITD and JIA in the general population should be probably stated and, in the discussion, those prevalences compared to the results obtained in the study population

-Prevalence of T1D and AITD in general population, were added both in the introduction and in the discussion, referring to the results obtained in the study population.

**RESULTS:**

**R:** Line 98 and 109, please clarify which subtype of JIA. Consider the paper Naddei et al. J Ped Rheumatology 2022.

-The oligo-articular subtype of JIA was clearly added and the Naddei paper was considered and added to the references.

**R:** Line 116, atopic dermatitis is not a sign but a comorbidity. Only dermatitis herpetiformis is a sign of celiac disease.

-It was rearranged.

**R:** Line 120-124, in my opinion the stats should be included both in the figure/table and in the text. I don't think the stats should be all included at the end of the section, in contrast this should be included within the results section

- Stats were embedded in the text and included also in the figures.

**R:** Please consider to exclude post-prandial hypoglycemia as a relevant symptom as it was not a clinical presentation of CD

-Post-prandial hypoglycaemia was excluded as relevant CD symptom following the referee suggestion.

**DISCUSSION**

**R:** Line 134, we believe that time at onset of autoimmunity other than celiac disease is crucial and must be indicated for each of the two groups and discussed. In fact, if time of follow-up in one group is longer that the latter, than also the chances to develop autoimmune condition may be different.

-Autoimmune conditions were evaluated at the onset of CD in both groups. We haven’t data on follow up period because the study is retrospective.

**R:** Line 137-138, we believe this should be discussed with reference to the overall prevalence of T1D, which is higher in M than F in the general population so the data are concordant with the literature.

-As suggested by the referee a sentence referring to IDDM prevalence in the male general population was added.

**R:** Line 144, sJIA or JIA, please specify and adjust the introduction accordingly.

- sJIA was corrected in JIA.

**R:** Line 146-147, there is no clear evidence in the literature indicated that higher levels of TTG in the serum are the consequence of a longer time between disease onset and diagnosis (i.e. a sort of cumulative effect of antibodies production). In contrast, high TTG titers well correlate with the presence of villous atrophy, this is the reason why with very high levels of TTg we can skip the duodenal biopsy assessment.

- Sentences were modified as suggested and the tone lowered.

**R:** Line 153-153, where is this piece of data shown? Please include a reference to a table or a figure in which the data is included.

- reference to figure 4 was added.

**R:** Line 183-185, in none of the ESPGHAN guidelines it is stated that you should wait until having very high levels of TTG to diagnose celiac. In fact for lower TG2 levels there is the indication to perform EGDS and obtain duodenal biopsy to assess whether villous atrophy is present or not.

-No one of our patients waited until having very high levels of anti-TTG antibodies, in order to receive diagnosis of CD. Nevertheless, a very high title of anti TTG IgA antibodies may be related to a longer length of disease also associated to a more advanced histological damage.

**R:** Line 187-189, the role of the genetic (male) sex onto the onset of additional AID could not be evaluated in the study as the cohort is small, so please edit the discussion accordingly.

-The discussion was modified according to the referee’s suggestions.

**FIGURES and TABLES**

**R:** I would rather combine what you call Table 1 and 2 (although I believe those are figures and not tables) to compare the sex distribution not only within each group but across groups and possibly include stats to evaluate differences between the 2 groups. Also Figure 1 and 2 should be merged and should be compared including the statistical tests performed (like in a table maybe?)

-Tables and figures were combined and merged according to referee’s suggestions. Figure 3 was added in order to compare the sex distribution related to prevalence of autoimmune disorders in the two groups as suggested by the reviewers.

English language was revised.

Best Regards.

Salvatore Accomando MD
